# Development of Tofun: A new sweet confection made from soymilk and honey

**DOI:** 10.1016/j.heliyon.2022.e10454

**Published:** 2022-08-28

**Authors:** Yasuhiro Arii, Kaho Nishizawa

**Affiliations:** a Department of Innovative Food Sciences, School of Food Sciences and Nutrition, Mukogawa Women's University, Nishinomiya, Hyogo, 663-8558, Japan; b Research Institute for Nutrition Sciences, Mukogawa Women's University, Nishinomiya, Hyogo, 663-8558, Japan

**Keywords:** Coagulation, Dysphagia, Honey, Novel-sweet development, Tofu processing

## Abstract

Tofu, a common part of the Asian diet, is soft, making it a good candidate for developing foods for people with dysphagia. Honey can coagulate soymilk proteins due to high levels of gluconic acid. In the present study, we aimed to investigate the soymilk-coagulating ability of 19 honey varieties to develop a soft sweet for people with dysphagia. We found that honey gathered from flora on Awaji Island had a higher coagulating ability than those of 18 other tested honey varieties. Based on the volume-to-weight ratio of honey, trial products were processed at honey concentrations of 20%, 25%, and 30% (w/w). The hardness, adhesiveness, and cohesiveness of products were examined according to the Consumer Affairs Agency's permissible criteria for foods for people with dysphagia. The hardness and adhesiveness of products at 25% and 30% honey concentrations were significantly higher than those at 20% honey concentration. Overall, the product evaluation was consistent with Permission Criteria II (can be swallowed after slight maceration). In terms of physical properties and product cost, the added honey concentration was determined to be 25% (w/w) for commercialization. The sweet was named “Tofun” and commercialized via collaboration with a local company as part of our community development efforts. The introduction of Tofun will encourage further innovation in the sugar confectionery industry.

## Highlights

• A new sweet “Tofun” was prepared using soymilk and honey
• Honey from Awaji Island has a high ability to aggregate soymilk
• The suitable honey concentration for processing Tofun was 25% (w/w)
• Tofun can be used as food for people with dysphagia

## 1 Introduction

Since ancient times, honey has been used as a natural remedy and in many foods owing to its characteristic flavor, taste, and texture. Recently, it has been attracting increasing attention as a superfood (Carina et al., 2014; Khan et al., 2018). Interestingly, honey can coagulate soymilk proteins, which is used to make tofu (Arii and Nishizawa, 2020). The ability of honey to coagulate soymilk proteins depends on the concentration of gluconic acid (GA), which is the coagulating agent (Arii and Nishizawa, 2020). The GA content ranges between 1.3 mg/g and 10.8 mg/g depending on the type of honey (Nishizawa et al., 2021). The mechanism of coagulation is similar to that observed in tofu formation due to the addition of glucono-δ-lactone (GDL), which changes to GA via hydrolysis, causing a decrease in the pH of soymilk (Nakayama et al., 1965; Chen et al., 2016; Arii et al., 2021). This reduces the electrostatic repulsion between soymilk proteins, leading to isoelectric precipitation and protein gel formation (Cavallieri and da Cunha, 2008; Arii et al., 2021). Moreover, honey is used for manufacturing sweets due to the presence of large quantities of sugars, predominantly fructose and glucose (Tewari and Irudayaraj, 2004). Additionally, honey exhibits antibacterial activity (Masoura et al., 2020; Majtan et al., 2021), and it affects the composition and activity of intestinal microflora in piglets (Biagi et al., 2006). This evidence strongly suggests that novel sweets can be prepared by adding honey to soymilk, which can help to maintain human health.

The amount of honey added, is a chemically and economically important factor for the development of novel sweets (Arii and Nishizawa, 2020). An excessive amount of honey results in candies that are too sweet and expensive whereas a deficient quantity added to soymilk does not result in coagulation. Therefore, honey with a high GA concentration is desirable for the development of sweets based on coagulated soymilk. The GA content significantly varies among honey produced under different conditions, and it negatively correlates with the total sugar content (Nishizawa et al., 2021). A previous study showed that the GA content of honey from Awaji Island (Hyogo, Japan) was the highest among those of all tested honey varieties (Nishizawa et al., 2021). It was approximately 8.3 times higher than that of honey with the lowest GA content. Thus, honey from Awaji Island has been deduced to have a high ability to coagulate soymilk.

The commercial use of tofu as a common, affordable, and adaptable food source makes it suitable for the development of novel sweet products. Most importantly, it is very soft (Yang and James, 2013). The characteristic softness makes it desirable to prepare new processed foods that can be eaten by people with difficulty in swallowing. Food choices for people with dysphagia (difficulty in swallowing) are understudied and limited, which contributes to their low quality of life. Thus, the development of new, widely available, palatable, and affordable food sources for people with such difficulties is needed, which will help to widen the range of food choices for them and improve their quality of life. Innovations in tofu processing and the confectionery industry will be useful in this regard.

The present study aimed to develop a practicable product by adding honey to soymilk. We investigated the coagulative ability of honey procured from Awaji Island on soymilk, and we prepared trial products by adding different concentrations of honey to soymilk. To explore the possibility of expanding food choices for people with dysphagia, textures of trial products were measured via a texture profile analysis test (Pons and Fiszman, 1996; Rosenthal, 2010) and evaluated against the Permission Criteria I, II, and III for foods for persons with dysphagia established by the Consumer Affairs Agency of Japan (2018). Furthermore, to demonstrate the utility of this scientific finding, we developed new sweets with a local small-to-medium-sized company.

## 2 Materials and methods

### 2.1 Materials

Polyfloral honey gathered by the Japanese honeybee (*Apis cerana japonica*) in spring on Awaji Island (Hyogo, Japan) was obtained from Heartoss Food Creates Co., Ltd. (Hyogo, Japan). Eighteen varieties of honey (designated as honey samples 1–18) were purchased from several vendors (Table 1). Ingredient-free soymilk (50 mg/mL protein) was purchased from Sujahta Meiraku (Aichi, Japan).

**Table 1:** Eighteen varieties of honey tested along with the honey from Awaji Island.

Honey	Floral source	Vendor^a^
1	Coffee	Yamada Bee Company, Inc.
2	Jarrah	The House of Honey
3	Cherry	Yamada Bee Company, Inc.
4	Manuka	Yamada Bee Company, Inc.
5	Blueberry	Yamada Bee Company, Inc.
6	Orange	Yamada Bee Company, Inc.
7	Lavender	Yamada Bee Company, Inc.
8	Sunflower	Yamada Bee Company, Inc.
9	Japanese Prickly-ash	Yamada Bee Company, Inc.
10	Japanese Bindweed	Yamada Bee Company, Inc.
11	Acacia	Yamada Bee Company, Inc.
12	Rosemary	Yamada Bee Company, Inc.
13	Linden	Yamada Bee Company, Inc.
14	Clover	Yamada Bee Company, Inc.
15	Lotus	Yamada Bee Company, Inc.
16	Hairy Vetch	Yamada Bee Company, Inc.
17	Polyflora	Kato Brothers Honey Co., Ltd.
18	Polyflora	Kanno Yohoen Honpo, Co., Ltd.

a Yamada Bee Company, Inc.; The House of Honey; Kato Brothers Honey Co., Ltd.; and Kanno Yohoen Honpo, Co., Ltd. are in Okayama, Japan; Herne Hill, WA, Australia; Tokyo, Japan; and Hyogo, Japan, respectively.

### 2.2 Preparation of diluted honey solutions for detection of coagulation

Diluted honey solutions were prepared using the method described in our previous report (Arii and Nishizawa, 2020). Briefly, diluted honey solutions were prepared by adding honey in distilled water at concentrations of every 10% from 0% (only distilled water) till 100% (v/v) (honey without distilled water).

### 2.3 Detection of the coagulative ability of honey

The ability of honey to coagulate soymilk proteins was detected using a previously reported method (Arii and Nishizawa, 2020). Soymilk was incubated at 85 °C for 5 min. Diluted honey solutions were mixed with an equal volume (0.5 mL) of incubated soymilk. Thereafter, the mixture was incubated at 85 °C for 60 min and then on ice for 60 min. The sample was separated into liquid and solid phases via centrifugation at 8,000 × *g* and 4 °C for 10 min. The layers in the mixture were visually observed. The separation of layers was used as a criterion for coagulation according to our previous study (Arii and Nishizawa, 2020).

### 2.4 Preparation of trial products

Considering the result of the coagulative ability experiment, honey was mixed thoroughly with soymilk to obtain 20%, 25%, and 30% (w/w) solutions at 25 °C. The respective mixtures were poured in jam bottles (Type 1, AS ONE, Osaka, Japan) with an inner diameter of 40 mm. The weight of the poured mixture was 25 g. Each bottled mixture was steamed on a low flame for 60 min, and this was followed by incubation on ice for 60 min. The products were stored at 4 °C overnight.

### 2.5 Measurement of the texture of trial products

The hardness, adhesiveness, cohesiveness, and thickness of trial products were measured using a texture profile analysis test (Pons and Fiszman, 1996; Rosenthal, 2010) with some modifications in the texture mode using a rheometer (RE2-33005S, Yamaden Co. Ltd., Tokyo, Japan). The jam bottle used to store trial products had the following specifications: plunger diameter of 20 mm, clearance of 5 mm, test speed of 1 mm/s, and temperature of 20 °C. The data represent the mean ± standard deviation values from three independent experiments.

### 2.6 Evaluation of the texture of sweet products

The textural quality was evaluated against the Permission Criteria I (2.5 × 10^3^–1 × 10^4^ N/m^2^ for hardness, 4 × 10^2^ J/m^3^ or less for adhesiveness, and 0.2–0.6 for cohesiveness), II (1 × 10^3^–1.5 × 10^4^ N/m^2^ for hardness, 1 × 10^3^ J/m^3^ or less for adhesiveness, and 0.2–0.9 for cohesiveness), and III (3 × 10^2^–2 × 10^4^ N/m^2^ for hardness, 1.5 × 10^3^ J/m^3^ or less for adhesiveness, and no standards for cohesiveness) for foods for persons with dysphagia established by the Consumer Affairs Agency of Japan (2018). Criteria I indicates that the food can be swallowed intact, such as a homogeneous jelly-like substance. Criteria II indicates that the food can be swallowed after it has been slightly pulverized in the mouth, e.g., pudding or mousse-like substances; however, it excludes those that conform to Criteria I. Criteria III includes heterogeneous foods that can be swallowed after little chewing, e.g., soft paste-like substances; however, it excludes foods that conform to Criteria I or II.

### 2.7 Statistical analysis

To quantitatively compare the textural values of trial products, one-way analysis of variance was performed for multiple-group comparisons, and this was followed by the Tukey honest significant difference test for post hoc analysis using KareidaGraph ver. 5.0 (Hulinks, Tokyo, Japan). Differences were considered statistically significant at *p*-values < 0.05.

## 3 Results and discussion

### 3.1 Precipitate formation by the addition of honey from Awaji Island

**Figure 1:**
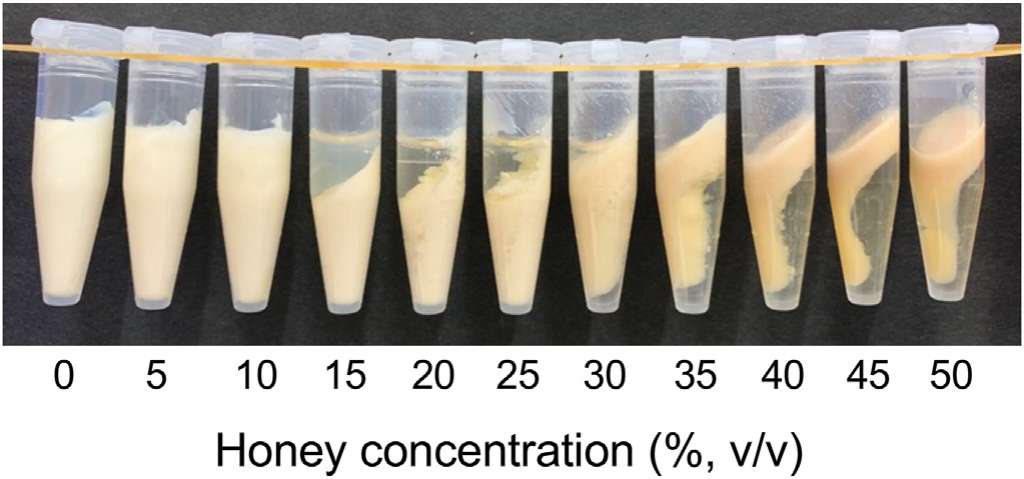
Concentration-dependent coagulation of soymilk using honey from Awaji Island.

**Figure 2:**
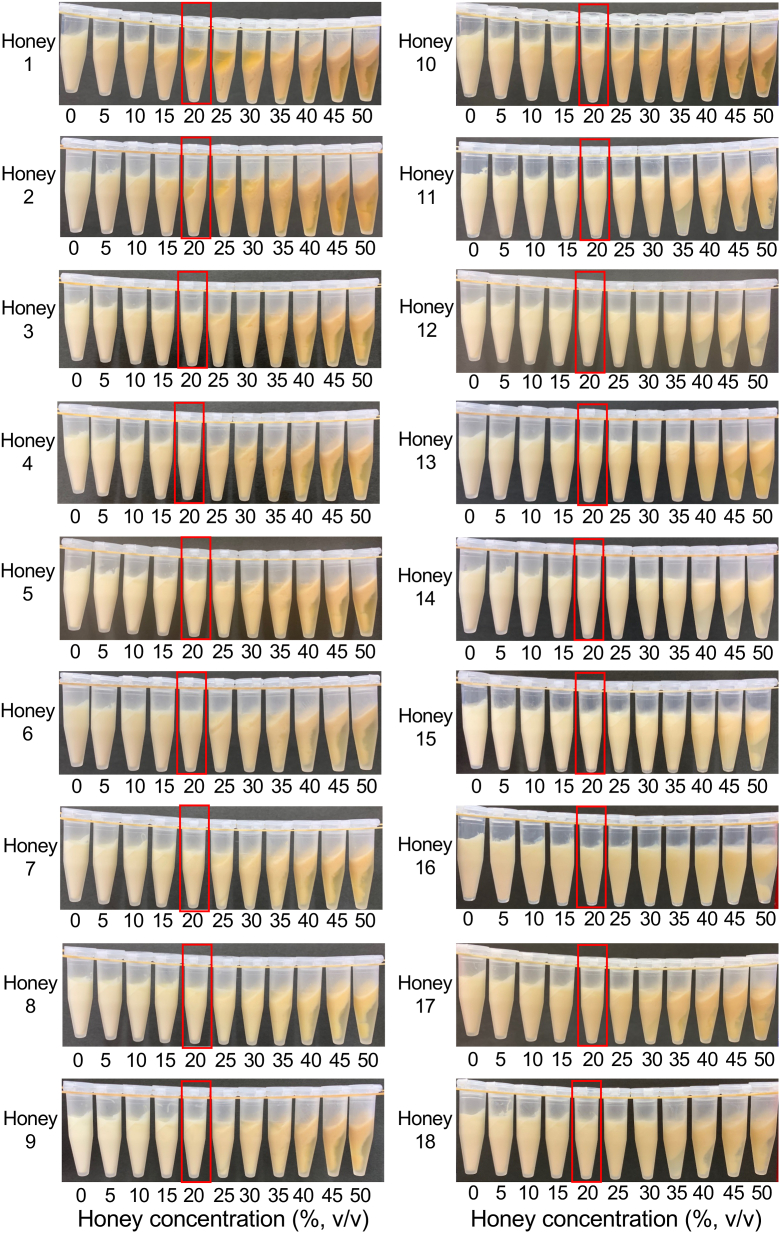
Concentration-dependent coagulation of soymilk using 18 other honey varieties. Honey samples 1–18 are indicated in Table 1. The red frames denote a honey concentration of 20%.

To investigate the ability of honey from Awaji Island to coagulate soymilk, diluted solutions of honey at concentrations ranging from 15%–25% (v/v) were added to soymilk, and the precipitates were observed (Figure 1). At concentrations above 30% (v/v), aggregates appeared in the upper layers. A previous study reported that precipitation occurs at low concentrations and aggregates appear in the upper layer at high concentrations when treated with honey containing a high concentration of GA (Arii and Nishizawa, 2020). The relationship between concentration and precipitate formation was consistent with that reported by Arii and Nishizawa (2020). Additionally, aggregates appear in the upper layer because the density of the precipitate is lower than that of the solution when the sugar content is high (Arii and Nishizawa, 2020). The other 18 honey varieties were also tested for the ability to coagulate soymilk (Figure 2). Honeys 1 and 2 induced soymilk aggregation at a concentration of 20%. In honeys 3–18, the soymilk did not separate into precipitates, and the honey concentrations in the supernatants were less than 20% (v/v). These results indicate that honey from Awaji Island had the highest ability to coagulate soymilk as compared to other varieties of honey. Our results correspond with those from previous studies, which have shown that the ability of honey to coagulate soymilk depends on the GA concentration (Arii and Nishizawa, 2020) and that honey from Awaji Island contains the highest amount of GA (10.8 mg/g honey) (Table 2), as reported in a previous study that examined the same varieties of honey that were used in the current study (Nishizawa et al., 2021). Of the organic acids present in small quantities in honey (<0.5%), GA is the major organic acid (>79%) (Mato et al., 2006). The presence of other organic acids implies that effective coagulation may not depend only on the GA content. Therefore, it is important to add honey to soymilk at various ratios and directly observe coagulation to select honey for further use, rather than only measuring their GA contents. GA is produced from glucose through a dehydrogenation reaction catalyzed by glucose oxidase, which is secreted from the hypopharyngeal gland of worker honey bees and participates in the maturation of nectar to produce honey (Takenaka et al., 1990; Ramachandran et al., 2006). The glucose oxidase reaction is also related to the antibacterial activity of honey (Masoura et al., 2020; Majtan et al., 2021). Measuring the antibacterial activity of honey has also attracted attention as a new qualitative standard (Majtan et al., 2021). In addition, the production of GA in honey is affected by various factors, including heating, the presence of catalase, and the presence of metal ions (Kleppe, 1966; Greenfield et al., 1975; Liu et al., 2001; Masoura et al., 2020). These reports have indicated that the storage conditions of honey may affect the coagulation of soymilk. Based on this evidence, a novel trial product was created by adding honey from Awaji Island to soymilk.

**Table 2:** Gluconic acid (GA) contents of the tested honeys.

Honey	GA content^a^ (mg/g of honey)
Awaji Island	10.8 ± 0.6^b^
1	4.3 ± 0.1^b^
2	4.8 ± 0.1
3	4.4 ± 0.1^b^
4	5.2 ± 0.2^b^
5	3.5 ± 0.1^b^
6	4.6 ± 0.1^b^
7	4.0 ± 0.1^b^
8	3.2 ± 0.1^b^
9	3.5 ± 0.0^b^
10	3.9 ± 0.1^b^
11	2.5 ± 0.0^b^
12	2.1 ± 0.0^b^
13	3.3 ± 0.1^b^
14	1.6 ± 0.0^b^
15	1.4 ± 0.0
16	1.3 ± 0.0^b^
17	2.4 ± 0.0^b^
18	2.5 ± 0.0^b^

a GA contents were analyzed using a commercially available GA assay kit, as described in Nishizawa et al. (2021).

b Data from Nishizawa et al. (2021).

### 3.2 Processing of a trial product using honey from Awaji Island

**Figure 3:**
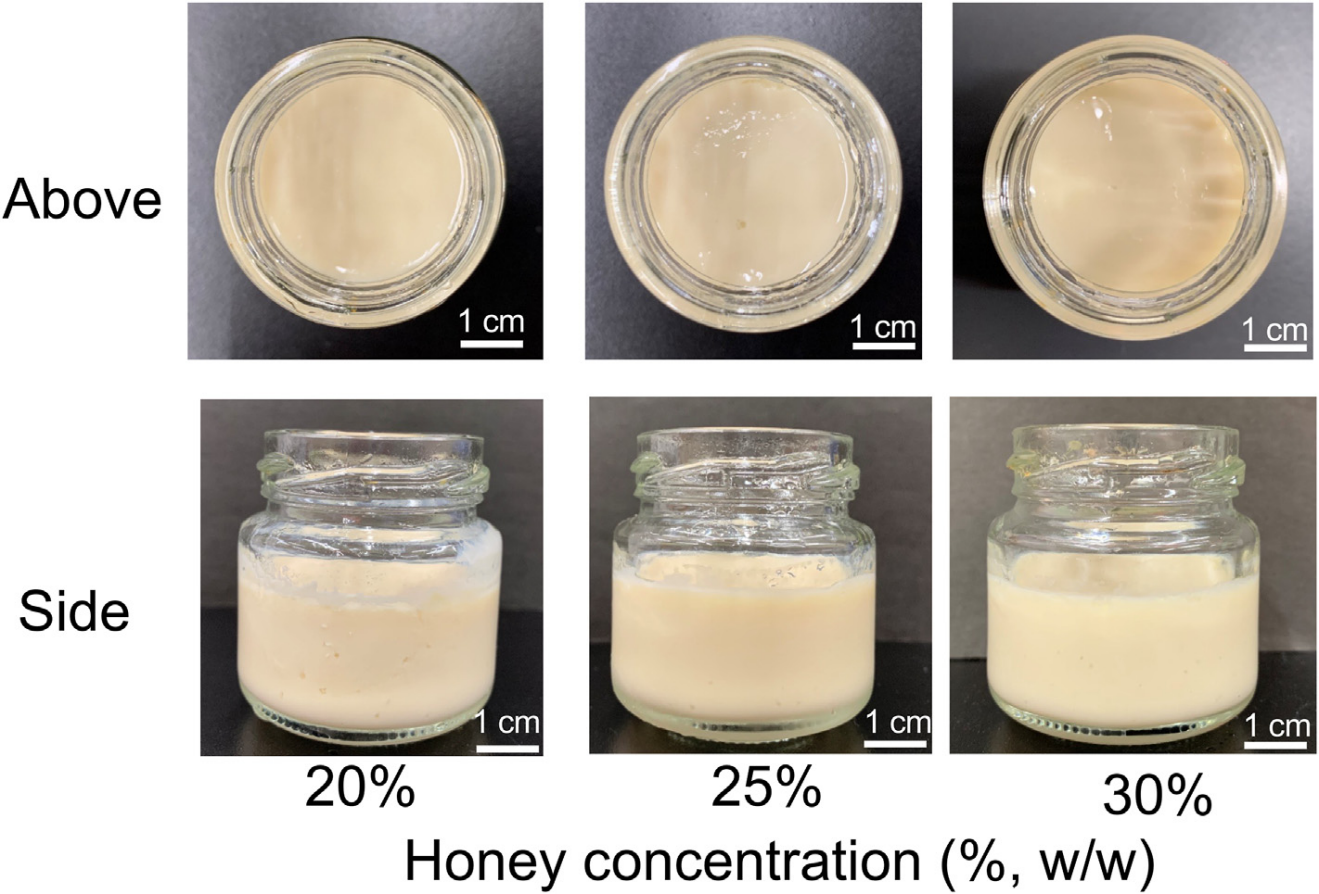
Trial products made from honey and soymilk. The mixture was poured into a jam bottle, steamed, and cooled.

The weights of 1 mL of honey from Awaji Island and soymilk were 1.38 g and 1.02 g, respectively. Based on the volume-to-weight ratio, a concentration of 15% (v/v) was calculated to be approximately 20% (w/w). In this honey, the GA concentration was roughly estimated to be 11 mM, which corresponded to the minimum concentration of GDL-coagulated soymilk (Arii et al., 2021). Hence, the minimum concentration of added honey to make the trial product should be greater than 20% (w/w). Additionally, as reducing the amount of honey leads to lower product cost (Aliaño-González, 2019), the maximum honey concentration was set at 30% to enable ease and effectiveness of practical applications. Soymilk was coagulated by adding honey at all tested concentrations (20%, 25%, and 30%; Figure 3). The results indicated the promising prospects of processing novel sweets practically by simply mixing honey with soymilk. Note that adequate heating was an important factor in soymilk coagulation during the processing of the trial products.

### 3.3 Textural analysis of trial products

The hardness, adhesiveness, and cohesiveness of trial products were measured (Table 3). The hardness and adhesiveness values of the trial product at a honey concentration of 20% (w/w) were significantly lower than those of the products with honey concentrations of 25% (*p* < 0.001 for hardness; *p* = 0.011 for adhesiveness) and 30% (w/w) (*p* < 0.001 for hardness; *p* = 0.016 for adhesiveness). No significant difference was observed between the products with honey concentrations of 25% and 30% in terms of hardness (*p* > 0.5) or adhesiveness (*p* > 0.5), although the hardness and adhesiveness values of products with 25% (w/w) honey concentration tended to be higher than those with 30% (w/w) honey. These results indicated that the trial product at 20% (w/w) honey concentration was softer and less sticky than that at higher concentrations. In terms of their cohesiveness values, no significant difference (*p* = 0.182 in 20% vs. 25%; *p* = 0.219 in 20% vs. 30%; *p* > 0.5 in 25% vs. 30%) was observed between the products at any of the concentrations. The results suggested that the ability of trial products crushed by the tongue to bind together to form a lump that is easy to swallow based on different honey concentrations, did not differ. However, it should be noted that these textures do not necessarily correspond with those perceived by humans (Funami, 2012).

**Table 3:** Textural analysis of trial products.

Honey concentration (%, w/w)	Hardness (×10^3^ N/m^2^)	Adhesiveness (×10^2^ J/m³)	Cohesiveness	Thickness (mm)
20	1.20 ± 0.08^a^	2.10 ± 0.25^a^	0.52 ± 0.03^a^	15.17 ± 0.15^a^
25	2.12 ± 0.06^b^	3.24 ± 0.28^b^	0.43 ± 0.02^a^	14.94 ± 0.14^a^
30	2.09 ± 0.08^b^	3.15 ± 0.25^b^	0.45 ± 0.02^a^	15.09 ± 0.09^a^

Data are expressed as the mean ± standard deviation of three independent experiments. Statistically significant differences were determined using one-way analysis of variance and the Tukey honest significant difference test. Means with different superscript letters in the same column indicate significant differences (*p* < 0.05).

### 3.4 Texture evaluation for foods for persons with dysphagia

The thickness of all the trial products was approximately 15 mm (Table 3). The measurements and texture values satisfied the permissible criteria for foods for persons with dysphagia established by the Consumer Affairs Agency of Japan (2018). Permission Criteria I must meet a hardness of 2.5 × 10^3^–1.0 × 10^4^ N/m^3^, an adhesiveness of less than 4 × 10^2^ J/m^3^, and a cohesiveness of 0.2–0.6. Permission Criteria II must meet a hardness of 1.0 × 10^3^–1.5 × 10^4^ N/m^3^, an adhesiveness of less than 1.0 × 10^3^ J/m^3^, and a cohesiveness of 0.2–0.9. Permission Criteria III must meet a hardness of 3.0 × 10^2^–2.0 × 10^4^ N/m^3^ and an adhesiveness of less than 1.5 × 10^3^ J/m^3^. The values of hardness, adhesiveness, and cohesiveness fell within the range of Permission Criteria II, I, and I, respectively, at all tested honey concentrations. Overall, the samples adhered to Permission Criteria II, which indicated that they could be swallowed after slight maceration in the mouth. The results strongly indicated that the products made from coagulating soymilk with honey sourced from Awaji Island can be used as food for persons with dysphagia.

Park et al. (2020) described that adhesiveness is the most important property of semi-solid foods for older adults with dysphagia. Food with a lower adhesiveness value is easier to swallow. In their study, foods such as whipped cream (8.7 × 10^2^ J/m^3^), soft tofu (7.2 × 10^2^ J/m^3^), and mango pudding (1.0 × 10^2^ J/m^3^) had low adhesiveness values. As shown in Table 3, adhesiveness values for our trial products were lower than that for soft tofu and higher than that for mango pudding. These results indicate that the trial products will be easier to swallow than soft tofu and harder to swallow than mango pudding.

### 3.5 Commercialization

**Figure 4:**
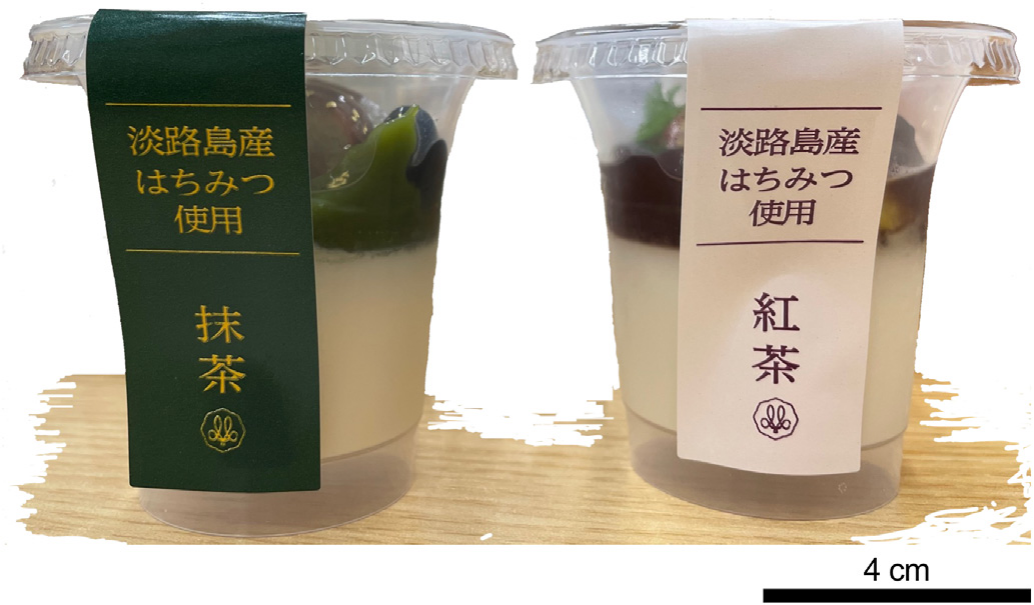
New sweet products made from honey and soymilk. The left image shows a product layered with green tea-flavored jelly and topped with the following sweetened ingredients: Japanese chestnuts, sweet potatoes, black soybean, and azuki beans. The right image shows a product layered with Earl Grey-flavored jelly and topped with orange.

To the best of our knowledge, there are no reports on the commercialization of sweets made from the coagulation of soymilk by the addition of honey. Hence, to demonstrate the utility of this scientific finding, a trial product was commercialized by collaborating with Heartoss Food Creates (Hyogo, Japan). Honey was mixed with soymilk at a concentration of 25% (w/w), which resulted in the hardest product, as shown in Table 4. The product obtained with 20% (w/w) honey concentration was soft and easily misshapen. It was also sweet and tasted like milk tea. The taste and flavor of the product were highly dependent on the type of honey used; thus, if the type of honey is changed, the taste and flavor also change. This feature is interesting from a business standpoint, as it allows for regional characteristics to be produced depending on the type of honey used. The new sweet was named "Tofun", which is a Chinese expression of a combination of the kanji meaning of "bean" and "bee", and it was prepared as two types of commercialized products (Figure 4). One commercialized product was layered with a green tea-flavored jelly on Tofun and topped with the following sweetened ingredients: Japanese chestnuts, sweet potatoes, black soybean, and azuki beans. The other product was layered with an Earl Grey-flavored jelly on Tofun and topped with orange. Tofun alone is tasty; however, honey is expensive, so the prices of products made with Tofun alone will be higher. Therefore, in order to reduce the price, increase volume, and create a sense of luxury, the abovementioned products were developed. In addition, the jelly layer also served to reduce the breakdown of Tofun during transport. The energy, protein, lipid, and carbohydrate contents and sodium chloride equivalent were calculated using the Standard Tables of Food Composition in Japan (MEXT, 2015), and they are summarized in Table 5. These products are new low-calorie sweets that are priced at 480 yen in Japan. This is the first report on the commercialization of a sweet, Tofun, prepared by coagulating soymilk with honey. The development of a product of this nature is a major innovation in the sweet industry.

**Table 4:** Evaluation of trial products for suitability for persons with dysphagia^a^

Honey concentration (%, w/w)	Hardness	Adhesivenss	Cohesiveness	Overall evaluation
20	II^b^	I^b^	I^b^	II^b^
25	II^b^	I^b^	I^b^	II^b^
30	II^b^	I^b^	I^b^	II^b^

a Data from Table 3 were evaluated for food for persons with swallowing difficulties established by the Consumer Affairs Agency of Japan (2018).

b Determination of Permission Criteria is as follows. Permission Criteria I must meet a hardness of 2.5 × 10^3^–1.0 × 10^4^ N/m^3^, an adhesiveness of less than 4 × 10^2^ J/m^3^, and a cohesiveness of 0.2–0.6. Permission Criteria II must meet a hardness of 1.0 × 10^3^–1.5 × 10^4^ N/m^3^, an adhesiveness of less than 1.0 × 10^3^ J/m^3^, and a cohesiveness of 0.2–0.9. Permission Criteria III must meet a hardness of 3.0 × 10^2^–2.0 × 10^4^ N/m^3^ and adhesiveness of less than 1.5 × 10^3^ J/m^3^.

**Table 5:** Energy, protein, lipid, carbohydrate, and sodium chloride content of commercialized sweet products made from honey and soymilk.

Tofun flavor	Energy^a^ (kcal, kJ)	Protein^a^ (g)	Lipid^a^ (g)	Carbohydrate^a^ (g)	Sodium chloride^a^ (g)
Green tea	66, 276	1.9	0.7	10.6	0.0
Earl Grey tea	51, 213	1.4	0.5	8.7	0.0

a The values were calculated as per-unit values using the Standard Tables of Food Composition in Japan (Ministry of Education, Culture, Sports, Science and Technology, Japan).

The currently available commercialized sweets have toppings that people with dysphagia find difficult to eat. This warrants the development of commercialized sweets that persons with dysphagia can easily consume. Additionally, the currently available products are too expensive to be used for meals in old-age facilities. For these reasons, commercialized sweets are marketed as high-end sweets for health-conscious people. However, the present study shows that the application of tofu processing could expand the food choices for people with dysphagia. In the near future, we hope to use the knowledge and experience gained in this study to develop varied food products for persons with dysphagia at a reduced cost.

## 4 Conclusion

Honey from Awaji Island has the highest ability to coagulate soymilk among the tested varieties of honey. In this study, we succeeded in developing a healthy and innovative sweet. The trial product, developed in collaboration with a commercial sweet company by adding honey to soymilk, was named Tofun. Tofun had a texture that made it suitable to be used as a food for people with dysphagia. We believe that such products will widen the range of food choices for persons with dysphagia and help improve their quality of life. Future research will be directed towards developing more cost-effective ways to create sweets or other confectioneries using natural products, for people with specific health conditions such as dysphagia.

## Declarations

### Author contribution statement

Yasuhiro Arii: Conceived and designed the experiments; Performed the experiments; Analyzed and interpreted the data; Contributed reagents, materials, analysis tools or data; Wrote the paper. Kaho Nishizawa: Performed the experiments; Analyzed and interpreted the data; Contributed reagents, materials, analysis tools or data.

### Funding statement

This work was supported by the JSBBA Academic Industrial R&D Support for Small-to-medium sized Enterprises, Tokyo, Japan.

### Data availability statement

Data will be made available on request.

### Declaration of interests statement

The authors declare the following conflict of interests: The authors; [have applied for a Japanese patent (Patent Application 2018-211706) for Tofun developed in this study].

### Additional information

No additional information is available for this paper.
